# Hemoglobin A1c measurement for the diagnosis of Type 2 diabetes in children

**DOI:** 10.1186/1687-9856-2012-31

**Published:** 2012-12-20

**Authors:** Chirag Kapadia, Philip Zeitler

**Affiliations:** 1 Phoenix Children’s Hospital, Phoenix, AZ 85016, USA; 2 Department of Pediatrics, University of Colorado, Denver, CO, USA

**Keywords:** Hemoglobin A1c, Type 2 diabetes, Pediatrics, OGTT, Retinopathy, Diagnosis

## Abstract

Laboratory measurements of hemoglobin A1c above 6.5% were approved as an additional diagnostic criteria for diabetes mellitus by the American Diabetes Association in 2010. Several recent pediatric studies have cast HbA1c measurement in children in an unfavorable light in the pediatric population, by comparing HbA1c measurements to results on oral glucose tolerance test (OGTT) or fasting plasma glucose (FPG). However, many of these studies do not recognize that diabetes diagnostic criteria are based upon long-term health outcomes. In this sense, OGTT and FPG have themselves never been validated in the pediatric population. Studies to validate diagnostic tests for diabetes in pediatric populations may take a substantial period of time, and may prove unfeasible. However, studies that tie diagnostic results as a child to diagnostic results as an adult may be more feasible and may provide the data needed to determine which pediatric diagnostic criteria to use. Thus, for the time being, except for cases of hemoglobinopathy, cystic fibrosis, and a few other exceptions, describing HbA1c as ‘lacking in sensitivity or specificity’ in the pediatric population because of lack of correlation with OGTT is not scientifically sound.

## Background

This paper is a commentary from the Pediatric Endocrine Society Drugs and Therapeutics Committee. Its goal is provide background information and guidance to both pediatric endocrinologists, and also to primary care practictioners on the current use of HbA1c for the diagnosis of Type 2 diabetes in children. In addition, it has the goal of pointing out some of the flaws in current methodologies being used to address this question.

Diagnostic criteria for diabetes were initially was based on variation from normal [1]. As information about the degrees of glycemia that lead to diabetic complications became available, criteria were revised. In 1997, the Expert Committee on Diagnosis and Classification of Diabetes Mellitus examined population data for retinopathy, and noted that for Fasting Plama Glucose (FPG), 2-hour postload glucose during an oral glucose tolerance test (OGTT), and hemoglobin A_1c_ (HbA1c), the diabetes-related complication of retinopathy increased linearly above a certain cutpoints; for FPG and OGTT, those cutpoints became the basis for the diagnosis of diabetes [2].

In 2010, it was felt that with the increasing adherence to the National Glycohemoglobin Standardization Program (NGSP), laboratory-based HbA1c is measured in a standardized fashion in the majority of labs in the U.S. Furthermore, the American Diabetes Association (ADA) noted that review of epidemiologic data supported a relationship between HbA1c and the risk of retinopathy similar to what had been shown for FPG and OGTT. Thus, in 2010, the ADA added A1c of 6.5 percent or greater as a diagnostic criterion [3].

The ADA required that a laboratory-based HbA1c assay method certified by the NGSP be used. This ensures that the assay used is standardized or traceable to the Diabetes Control And Complications Trial [4,5].

Obtaining screening laboratories for diabetes in the at-risk population has long been established practice in adults. However, in the pediatric population, data have thus far largely been extrapolated from adult studies, and screening practices vary. The issue has become more pertinent with the rise of pediatric obesity. While FPG and OGTT thresholds, as extrapolated from adult populations, have largely been accepted by the community of pediatric practictioners, the more recent recommendation of use of HbA1c has met resistance.

## Main text and discussion

An important benefit of use of HbA1c is that patient does not need to be fasting, and testing does not require a return visit [6]. HbA1c has less variability and is more reproducible than FPG and OGTT [7].

However, HbA1c also has several potential disadvantages. Hb A1c may miss cases of Type 1 diabetes in which hyperglycemia develops over a short period of time. Furthermore, HbA1c is not a perfect estimation of mean blood glucose, and varies by ethnicity [8,9]. In addition, diseases such as iron deficiency anemia, sickle-cell disease, thalassemia, and other hemoglobinopathies, can alter HbA1c [10].

## Recent studies

When FPG was used to diagnose diabetes, HbA1c of 6.5% had sensitivity of 75.0% and specificity of 99.9% [11]. The authors examined the diagnosis of pre-diabetes noted a low sensitivity but a good specificity (98.3%) for a HbA1c of 5.7%, and also for HbA1c of 6.0% (specificity 99.4%).

Another study [12] compared HbA1c to OGTT in over 1000 obese patients and concluded that A1c has low sensitivity and specificity for diabetes when diabetes is defined by OGTT results; 9 of 893 patients with an HbA1c less than 5.7% were determined to have diabetes using OGTT criteria. In addition, a larger number of cases of prediabetes defined by OGTT were not identified using an HbA1c cut-off of 5.7%; 240 of the 347 (69%) cases of prediabetes in this high-risk population had HbA1c <5.7%.

In a smaller study, HBA1c cut-off of 6.5% had a sensitivity of 40% and a specificity of 96% in accurately diagnosing patients with type 2 diabetes, when using OGTT as a gold standard [13].

A study of 254 overweight or obese adolescents also raised questions about HbA1c use for diagnosis [14]. In this study, there were 99 (39%) cases of prediabetes and 3 (1.2%) cases of diabetes using FPG and OGTT as gold standards. Test performance was assessed using receiver operating characteristic (ROC) curves and calculations of area under the ROC curve (AUC). HbA1c (AUC 0.54 [95% CI 0.47-0.61]) displayed poor discrimination for identifying children with dysglycemia that had been identified on OGTT. In fact, in this study, random glucose (AUC 0.66 [0.60-0.73]) had better correlation with OGTT.

The potential advantages of use of HbA1c, however, were born out by a study before and after a change in recommendation to allow use of HbA1c in screening of adolescents [15]. Rates of screening for diabetes increased from 39 to 47% as a result, and this led to twice as many incident T2DM diagnoses during a similar time period. HbA1c threshold of 6% indicated progression to diabetes in 18% of patients, while the 5.7% threshold resulted in only 1.3% progression to diabetes, over about 3 years . In a separate study with 468 subjects, HbA1c cut-off of 6% had greater correlation with OGTT results than A1c threshold of 5.7% [16]. This study showed sensitivity and specificity of 86% and 85%, respectively, for HbA1c threshold 5.7%, but 99% and 96%, respectively, for HbaA1c threshold of 6%.

### Comparing unvalidated methodologies: a pitfall of many studies

Studies that compare HbA1c to other methods of diagnosing diabetes in pediatric populations are handicapped by the fact that the other methods – FPG and OGTT – are themselves not validated in the pediatric population. A truly validated definition of diabetes in pediatric populations requires insight into the relationship of the proposed definitions to relevant aspects of medium and long-term health [17].

Assuming that OGTT or FPG are better than HbA1c for determining risk of complications is unfounded; in adults, even though HbA1c, FPG, and OGTT are often discrepant in individuals, all 3 markers are shown to correlate very well with risk of complications [2,3].

## Conclusions

The diagnostic thresholds of glycemia in the adult population were formulated because these are theh thresholds at which retinopathy increases. Such thresholds have never been defined in the pediatric population. In this sense, for diagnosis of diabetes in asymptomatic or minimally symptomatic children, there are no validated methodologies. OGTT, which is being used as a ‘gold standard’ in many studies, in addition to lacking validation as noted above, also suffers from having low reproducibility [18]. Therefore, dismissal of HbA1c 6.5% or greater for diagnosis of diabetes at this time, because of lack of correlation with OGTT, is a flawed approach.

The lack of correlation between studies has already been acknowledged and discussed in adult populations, including in the ADA’s clinical practice guidelines [3,4]. While the correlation appears to be lower in pediatrics, it is not yet clear which of the studies, if any, are ‘faulty,’ and obtaining such data would require comprehensive, multi-center, long-term studies on incidence of retinopathy; such studies appear unlikely to occur at this time.

Therefore, conclusions that dismiss HbA1c use for the diagnosis of diabetes in children are based on incomplete data. Considering that the demographics of Type 2 diabetes skew towards disadvantated populations, we should not dismiss a valuable, flexible tool that, put into widespread use, may in fact increase, not decrease, early detection of this disease [15].

### Recommendations

1. ADA criteria for the diagnosis of diabetes, though formulated from data in adults, are useful for screening the asymptomatic, or minimally symptomatic, at-risk pediatric population at this time, and this includes the ADA’s more recent recommendations regarding HbA1c.

2. When test results do not correlate with each other, or appear to give information conflicting to the clinical situation, practictioners must use clinical judgement. Amongst FPG, OGTT, or HbA1c, one test is not validated to a greater extent than another in the pediatric population.

3. There are some questions about HbA1c use in those with cystic fibrosis [19], though some data shows it may be useful [20].

4. HbA1c is likely reliable in those with sickle-cell carrier status, as long as an assay without interference from abnormal hemoglobins is used [5,21]. HbA1c is not reliable in those known to have a hemoglobinopathy or any other disorder resulting in significant increases in red blood cell turnover, or in pregnancy [22]. Assay interference information is available on the NGSP website [5].

5. Laboratory-based hemoglobin A1c using a methodology and assay certified by the National Glycohemoglobin Standardization Program is the preferred method for diagnostic purposes [3-5].

## Abbreviations

ADA: American Diabetes Association; FPG: Fasting Plasma Glucose; OGTT: Oral Glucose Tolerance Test; HbA1c: Hemoglobin A1c; NGSP: National Glycohemoglobin Standardization Program.

## Competing interests

There are no competing interest for any participating authors or collaborators.

## Authors’ contributions

CK and PZ are the primary authors and editors of this commentary. The remaining collaborators from the PES Drugs and Therapeutics Committee helped shape the article through discussion at meetings and conference calls, and provided editing of the manuscript. All authors read and approved the final manuscript.

## Authors’ information

Pediatric Endocrine Society Drugs and Therapeutics Committee for 2011–2012:

Kapadia C^a^, Zeitler P^b^, Divall, S^c^, Grimberg, A^d^, Gitelman SE^e^, Quintos JB^f^, Draznin M^g^, Nebesio T^h^, Myers SE^i^, Potter A^j^, Vogiatzi M^k^, Raman S^l^, Waguespack SG^m^, Eugster E^h^, Boney CM^f^, Rosenthal SM^e^, Silverstein J^n^

a. Phoenix Children’s Hospital, Phoenix, AZ

b. Department of Pediatrics, University of Colorado, Denver, CO

c. Johns Hopkins University, Baltimore, MD

d. Children’s Hospital of Philadelphia, Philadelphia, PA

e. University of California-San Francisco, San Francisco, CA

f. Rhode Island Hospital, The Warren Alpert Medical School of Brown University, Providence, RI

g. Michigan State University Kalamazoo Center for Medical Studies, Kalamazoo, MI

h. Riley Children’s Hospital, Indianapolis, IN

i. Cardinal Glennon Children’s Hospital, St Louis, MO

j. Vanderbilt University Medical Center, Nashville, TN

k. Weill Medical Center of Cornell University, NY, NY

l. Children’s Mercy Hospital, Kansas City, KS

m. University of Texas M.D. Anderson Cancer Center, Houston, TX

n. University of Florida College of Medicine, Gainesville, FL
